# Language Barriers and Other Determinants of Post–Intensive Care Unit Follow-Up: A 5-Year Review

**DOI:** 10.1016/j.mayocpiqo.2026.100694

**Published:** 2026-02-19

**Authors:** Ibrahim S. Karakus, Annie B. Johnson, Andrew C. Hanson, Kellie A. Robbins, Sumera R. Ahmad, Lioudmila V. Karnatovskaia, Amelia K. Barwise

**Affiliations:** a Division of Pulmonary, Critical Care, Allergy & Sleep Medicine, Mayo Clinic, Rochester, MN; b Department of Quantitative Health Sciences, Mayo Clinic, Rochester, MN; c Department of Anesthesiology and Perioperative Medicine, Mayo Clinic, Rochester, MN; d Biomedical Ethics Research Program, Mayo Clinic, Rochester, MN

## Abstract

**Objective:**

To evaluate factors associated with attendance at a post–intensive care syndrome (PICS) follow-up clinic, with a focus on language barriers and socioeconomic status.

**Patients and Methods:**

We conducted a retrospective cohort study at Mayo Clinic, Rochester, Minnesota, from January 1, 2019, through June 30, 2024. Adult patients (≥18 years) with an ICU stay of 3 days or more who were referred to the PICS clinic were included. We examined 2 levels of outcomes: attendance rates and clinical outcomes among attendees. Attendance was evaluated in relation to demographic and clinical characteristics, language proficiency, interpreter needs, and socioeconomic status measured by the housing-based socioeconomic status index. Among attendees, patient-reported outcomes were assessed using the EuroQol-5D and the posttraumatic stress disorder (PTSD) checklist for DSM-5.

**Results:**

Of 2001 referred patients, 943 (47.1%) attended the clinic. Attendance did not differ significantly by age, sex, race, or housing-based socioeconomic status index quartile but was lower among patients who had language barriers (31.1% vs 47.7%; *P*=.011) and those requiring interpreter services (29.4% vs 47.6%; *P*=.010). Longer ICU (≥11 days) or hospital stays (≥21 days) were associated with lower attendance. Among attendees, non-English-speaking patients and those requiring interpreters had markedly lower EuroQol-5D and PTSD Checklist for DSM-5 completion rates. In multivariable analysis, female sex, prolonged hospitalization, and unemployment were independently associated with worse quality of life and PTSD scores.

**Conclusion:**

Language barriers, interpreter needs, and prolonged ICU or hospital stays were associated with lower PICS clinic attendance. Language barriers also reduced engagement with recovery assessments. Targeted outreach, language support, and translated materials may improve attendance and the effectiveness of post-ICU follow-up care for these patients.

Post–intensive care syndrome (PICS) refers to a range of physical, psychological, and cognitive impairments that persist after hospital discharge and can occur as the aftermath of a prolonged intensive care unit (ICU) stay or critical illness experience, ultimately leading to delayed recovery and reintegration into social life.^1^, ^2^, ^3^ Approximately 6 million people are admitted to ICUs in the United States annually, and approximately 50% to 70% of ICU survivors experience at least 1 of the aforementioned impairments.^4^ Therefore, PICS represents a major health concern.^5^^,^^6^ To address the needs of this patient population, outpatient PICS clinics, also known as ICU recovery clinics or ICU follow-up clinics, have been established to follow-up with these patients after discharge.^7^ The PICS clinic is a multidisciplinary care model that aims to mitigate potential adverse outcomes of critical illness through cognitive assessments, nutritional counseling, pain management, and psychosocial support.^1^

Early follow-up after ICU discharge may speed up physical recovery, improve psychological well-being and quality of life, and prevent future adverse effects.^8^^,^^9^ However, few patients attend follow-up appointments in PICS clinics owing to a combination of patient-related, logistical, and system-level barriers. Key factors include physical and psychological limitations after critical illness, distance from the clinic, insufficient physical strength, age, sex, education levels, race, and socioeconomic status (SES). Furthermore, the digital divide or inequities in usage and access to digital services and lack of awareness about the existence or purpose of these clinics among patients and families, as well as among providers, contribute to low attendance.^9^, ^10^, ^11^, ^12^, ^13^, ^14^ Discharge documentation is often incomplete, leading to confusion about follow-up responsibilities and referral processes.^11^ System issues include inadequate funding, limited clinic capacity, and poor communication between ICU teams and community providers.^10^, ^11^, ^12^^,^^15^, ^14^, ^16^, ^17^

Language barriers may represent an additional challenge for patients. Evidence highlights that patients with language barriers face significant difficulties in accessing and using health care services generally, including outpatient follow-up after ICU discharge. Language barriers may delay access to care, interfere with understanding of discharge instructions, and reduce engagement with follow-up services as patients with language barriers are less likely to be provided with information they understand about the purpose of the follow-up appointments.^18^^,^^19^ Additionally, language barriers are associated with lower health literacy, reduced trust, and decreased patient engagement, which further decreases the likelihood of attending post-ICU clinics.^20^ However, language barriers and post-ICU follow-up clinic attendance have not been widely studied.^8^

The Mayo Clinic PICS clinic began accepting patients in 2019 and has been operating for 5 years. Our objective was to evaluate and describe the characteristics of patients who attended the clinic, as well as the factors contributing to nonattendance, with a particular focus on language barriers and SES measured by the novel housing-based socioeconomic status (HOUSES) index.^21^ We hypothesized that language barriers and socioeconomic background would be associated with lower clinical attendance. We also aimed to summarize the clinical outcomes evaluated during the post-ICU follow-up clinic.

## Patients and Methods

### Study Design and Setting

We conducted a retrospective cohort study at the Mayo Clinic, Rochester, Minnesota, a quaternary care academic medical institution with 2059 inpatient beds and 7 adult ICUs, with a total of 250 ICU beds. Inclusion criteria were as follows: (1) age 18 years or older, (2) ICU stay of 3 days or more, and (3) referral to PICS clinic following hospital discharge. We chose 3 days because PICS studies commonly define ICU exposure using 24-72 hours of ICU stay, which avoids short-term observational ICU admissions. It also aligns with our institution’s typical ICU length of stay (∼3 days); a longer cutoff (eg, 7 days) would exclude many otherwise eligible patients. This is also a standard length of stay used to refer patients to the PICS clinic.

Eligible patients are scheduled through the electronic health system. Automated reminders are sent through the patient’s electronic health record (EHR) 2 days before the visit. All visits operate every other Thursday and Friday, with seven 1-hour virtual consults scheduled per day via phone or video. Each visit is attended in full by an advanced practice provider, a pharmacist, and an occupational therapist. The multidisciplinary team takes a comprehensive approach to recovery by addressing physical, emotional, and cognitive symptoms; reconciling medications and follow-up plans; and assessing functional needs. Remote video interpretation is routinely used during clinic visits and includes a wide range of languages. Patient-reported outcome measures (EuroQol [EQ]-5D and posttraumatic stress disorder [PTSD] checklist for DSM [PCL]-5) are completed electronically; however, they are available only in English. Proxies may complete these questionnaires on the patient’s behalf, with proxy completion documented in the medical record.

We collected data from the EHR of patients referred to the PICS clinic between January 1, 2019, through June 30, 2024. The institutional review board (IRB) reviewed and approved the study as minimal risk (IRB-24-007470). Per our IRB, this retrospective study did not require consent. In accordance with Minnesota state law, patients who did not provide research authorization were excluded from the analysis. We report the results in accordance with the EQUATOR network’s STROBE (Strengthening the Reporting of Observational Studies in Epidemiology) statement.^22^

### Predictor Variables

Demographic variables included age, sex, race, ethnicity, preferred language, interpreter use and level of education. Medical complexity was assessed by illness severity scores including acute physiology and chronic health evaluation (APACHE) III score, Charlson score, and sequential organ failure assessment (SOFA) score. Additionally, we examined the type of ICU admission (eg, medical, surgical, trauma, and neurologic) and the length of ICU and hospital stays.

To assess patient SES, we used the HOUSES index developed by Dr Juhn at Mayo Clinic. This index serves as a robust individual-level SES measure based on 4 key housing characteristics—number of bedrooms, number of bathrooms, square footage, and estimated property value—obtained from local government assessor records.^21^^,^^23^, ^24^, ^25^, ^26^, ^27^, ^28^ Originally developed in Olmsted County, the HOUSES index is now available for use nationwide across all 50 states and has been linked to over 62 health care–related outcomes.^24^^,^^27^^,^^29^, ^30^, ^31^ The HOUSES index was standardized into quartiles, with Q1 representing the lowest SES and Q4 the highest.

### Clinical Attendance and Outcome Variables

The main outcome of interest was attendance at the PICS follow-up clinic. We examined patient-reported outcomes using the PTSD checklist for DSM (PCL)-5 survey for PTSD and EQ-5D survey for quality of life (Figure).

**Figure:**
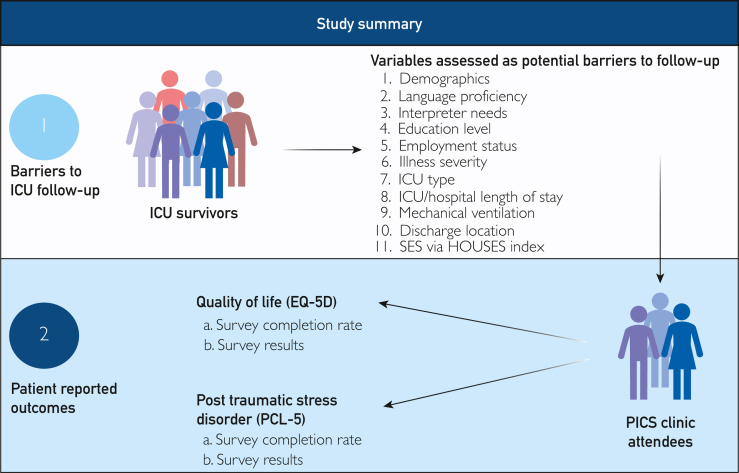
Summary of study method. Created in BioRender. Karakus S. (2026) https://BioRender.com/9iv14nj

Data collection was completed by an Anesthesia Clinical Research Unit Data Specialist using electronic data retrieval tools for large cohort coverage—Mayo Data Explorer and Department-Specific DataMarts—supplemented by manual chart review as needed. Mayo Data Explorer is a Mayo Clinic developed web application housing both structured and unstructured data, created using data from Mayo Clinic’s Unified Data Platform data warehouse environment. Department-Specific DataMarts are validated EHR-based infrastructure, housing ICU, odds ratio (OR), and Hospital Reporting DataMarts.

### Statistical Analyses

Data are summarized as frequency (percentage), with continuous variables categorized for presentation of PICS attendance and score ascertainment rates. Rates were presented as numbers (percentages) and were compared across categories using Pearson χ^2^ tests. Univariable proportional odds models were used to assess the association between characteristics and patient-reported outcomes, and results were reported as ORs with 95% CIs and *P* values. Multiple imputation with 100 multiply imputed data sets was used to account for missing data. The imputation model included ICU specialty, age, sex, English language, interpreter use and request, race, ethnicity, education, employment status, APACHE III, SOFA, Charlson comorbidity index, HOUSES, ICU and hospital length of stay, ventilator use and duration, discharge location, and EQ5Dand PCL components and total. Complete case analysis was performed as a sensitivity analysis. *P* values of <.05 were considered statistically significant. All analysis were done using the R software, version 4.4.1 (R Foundation for Statistical Computing).

## Results

### Referral Cohort Patient Demographic Characteristics

A total of 2001 patients were referred to the PICS clinic. The median (25th, 75th percentile) age was 63 (51, 71) years. A total of 1157 (57.8%) were men, and 88.6% were identified as White race. Patients with language barriers comprised 3.0% of our cohort (n=61); 51 (2.5%) needed an interpreter. The median ICU length of stay was 5.3 (3.9, 8.8) days, and the median hospital stay was 12 (8, 21) days. The median APACHE III severity of illness score was 91 (65, 122), and the median SOFA score was 8 (4, 12) (Table 1).

**Table 1:** Patient Demographic Characteristics

Characteristic	Overall (N=2001)
Age, y	63 (51-71)
Sex	
Female	844
Male	1157
Race	
White	1772 (88.6)
Black	75 (3.7)
Asian	50 (2.5)
American Indian/Alaska Native/Native Hawaiian	29 (1.4)
Other	75 (3.7)
Patients with language barriers	61 (3.0)
Interpreter use	51 (2.5)
Education (n=1546)	
Less than HS	97 (6.3)
HS graduate	502 (32.5)
Some college	491 (31.8)
4-y degree	321 (20.8%)
Postgraduate degree	135 (8.7)
ICU LOS, d	5.3 (3.9-8.8)
Hospital LOS, d	12 (8-21)
Invasive ventilation use	1328 (66.4)
APACHE III	91 (65-122)
SOFA	8 (4-12)
Discharge to home	1691 (84.5)
HOUSES index, quartile (n=1784)	
Q1	403 (22.6)
Q2	414 (23.2)
Q3	452 (25.3)
Q4	515 (28.9)
PICS attendance	943 (47.1)

Values are n (%) or median (IQR).

APACHE, acute physiology and chronic health evaluation; HOUSES, housing-based socioeconomic status; ICU, intensive care unit; LOS, length of stay; Q, quartile; SOFA, sequential organ failure assessment.

### PICS Clinic Attendance

Among the 2001 referred 943 (47.1%) attended the clinic. Attendance did not vary significantly across age groups, sex, or race ranging from 33.3% among Black patients to 54.0% among Asian patients (*P*=.14). Attendance was similar between those reporting Hispanic ethnicity and those not (44% vs 47.3%, respectively; *P*=.25). Although not statistically significant, attendance was numerically higher among patients with a 4-year degree than that among those reporting some college education or less (55.1% vs 48.7%; *P*=.22).

Patients with language barriers attended at significantly lower rates than English-speaking patients (31.1% vs 47.7%; *P*=.01). Similarly, patients requiring interpreter services had a significantly lower attendance rate compared to those who did not need an interpreter (29.4% vs 47.6%; *P*=.01).

Patients with longer ICU stays were less likely to attend the clinic (40.4% vs 48.6% among patients staying ≥11 days vs those staying less; *P*=.02). Similarly, patients with longer hospital stays were less likely to attend than those with shorter stays (39.6 vs 50.5% among those with 21 days or longer vs those <21 days; *P*<.001).

Use of invasive mechanical ventilation was not associated with PICS clinic attendance (47% among both groups; *P*=.94). Similarly, when stratified by duration of ventilation, there was no significant association with follow-up attendance (*P*=.301).

Patients discharged home showed higher attendance than those discharged elsewhere such as to a skilled nurse facility, assisted living, or hospice (48.0% vs 42.3%; *P*=.062). Illness severity as measured by APACHE III and SOFA scores as well as comorbidity burden as measured by Charlson index were not significantly associated with PICS clinic attendance. Socioeconomic status, as measured by the HOUSES index, was not significantly associated with PICS clinic attendance (*P*=.604) with similar rates across all quartiles (Table 2).

**Table 2:** Demographic, Clinical, and Socioeconomic Characteristics by PICS Attendance

Characteristic	Total (n)	Seen in PICS, n (%)	*P*
Age, y	2001		.34
0-49	444	193 (43.5)	
49-59	374	177 (47.3)	
59-69	611	299 (48.9)	
69-100	572	274 (47.9)	
Sex	2001		.803
Men	1157	548 (47.4)	
Women	844	395 (46.8)	
Race	2001		.14
White	1772	844 (47.6)	
Black	75	25 (33.3)	
Asian	50	27 (54.0)	
American Indian/Alaska Native/Hawaiian	29	13 (44.8)	
Other	75	34 (45.3)	
Language	2000		.01^a^
Non-English	61	19 (31.1)	
English	1939	924 (47.7)	
Interpreter needed	2001		.01^a^
No	1950	928 (47.6)	
Yes	51	15 (29.4)	
Invasive ventilation use	2001		.94
No	673	318 (47.3)	
Yes	1328	625 (47.1)	
APACHE III score	2001		.74
0-74	691	326 (47.2)	
75-109	620	285 (46.0)	
110+	690	332 (48.1)	
SOFA score	2001		.96
0-5	720	342 (47.5)	
6-10	593	277 (46.7)	
11+	688	324 (47.1)	
ICU length of stay, d	2001		.02^a^
0-4	925	450 (48.6)	
5-10	710	345 (48.6)	
11+	366	148 (40.4)	
Hospital length of stay, d	2001		<.001^a^
0-6	297	150 (50.5)	
7-20	1212	598 (49.3)	
21+	492	195 (39.6)	
HOUSES index, quartile (n=1784)	1784		.604
Q1	403	182 (45.2)	
Q2	414	192 (46.4)	
Q3	452	223 (49.3)	
Q4	515	249 (48.3)	
Discharge location	2001		.062
Other	310	131 (42.3)	
Home	1691	812 (48.0)	

APACHE, acute physiology and chronic health evaluation; HOUSES, housing-based socioeconomic status; PICS, post–intensive care syndrome; Q, quartile; SOFA, Sequential Organ Failure Assessment.

a Statistically significant.

### Patient-Reported Outcomes Among Attendees (Quality of Life and PTSD Evaluations)

Among patients who attended the PICS clinic, language barriers were significantly associated with lower completion rates of both the EQ-5D and PCL-5 questionnaires. Although the number of these patients who attended the clinic was small (n=19), their questionnaire completion rates were markedly lower than English-speaking patients (31.6% vs 74.2% for EQ-5D; *P*≤.001 and 21.1% vs 42.9% for PCL-5; *P*=.05). A similar trend was observed among patients who required an interpreter, with only 26.7% completing the EQ-5D and 20.0% completing the PCL-5, compared with 74.1% and 42.8%, respectively, among those who did not need an interpreter (*P*=.07). Additionally, lower educational attainment was associated with reduced PCL-5 completion, with rates ranging from 24.4% among those reporting less than HS degree to 70.3% in those reporting a postgraduate degree (*P*<.001) (Table 3).

**Table 3:** Survey Completion Rates by Patient Demographics and Socioeconomic Characteristics

Characteristic	EQ-5D completion, n (%)	*P*	PCL-5 completion, n (%)	*P*
Age, y		.141		.26
0-49	136 (70.5)		77 (39.9)	
49-59	137 (77.4)		86 (48.6)	
59-69	228 (76.3)		128 (42.8)	
69-100	191 (69.7)		109 (39.8)	
Sex		.669		.39
Men	405 (73.9)		226 (41.2)	
Women	287 (72.7)		174 (44.1)	
Race		<.001^a^		.19
White	637 (75.5)		369 (43.7)	
Black	12 (48.0)		8 (32.0)	
Asian	17 (63.0)		9 (33.3)	
American Indian/Alaska Native/Native Hawaiian	8 (61.5)		3 (23.1)	
Other	18 (52.9)		11 (32.4)	
Language		<.001^a^		.06
Non-English	6 (31.6)		4 (21.1)	
English	686 (74.2)		396 (42.9)	
Interpreter needed		<.001^a^		.08
No	688 (74.1)		397 (42.8)	
Yes	4 (26.7)		3 (20.0)	
Education		.324		<.001^a^
Less than HS	28 (62.2)		11 (24.4)	
HS graduate	170 (71.4)		88 (37.0)	
Some college	180 (75.3)		112 (46.9)	
4-y degree	132 (74.6)		96 (54.2)	
Postgraduate degree	50 (78.1)		45 (70.3)	
HOUSES index, quartile		.273		.064
Q1	127 (69.8)		77 (42.3)	
Q2	150 (78.1)		85 (44.3)	
Q3	162 (72.6)		86 (38.6)	
Q4	188 (75.5)		126 (50.6)	

EQ, EuroQol; HS, high school; HOUSES, housing-based socioeconomic status; PCL-5, posttraumatic stress disorder checklist for DSM-5; Q, quartile.

a Statistically significant.

In multiple imputation analysis, women were more likely to report PTSD symptoms (OR, 1.53; 95% CI, 1.15-2.03; *P*=.003) and less likely to report favorable quality of life (OR, 0.77; 95% CI, 0.60-0.99; *P*=.044). Hospital length of stay over 21 days was associated with lower quality of life (OR, 0.56; 95% CI, 0.37-0.86; *P*=.008). Furthermore, patients who were unemployed had significantly higher PTSD symptom scores than employed patients (OR, 3.77; 95% CI, 1.55-9.19).

## Discussion

In this retrospective cohort study of patients referred to the Mayo PICS clinic, we found that fewer than half of eligible patients attended follow-up. Attendance did not differ significantly by age, sex, race, ethnicity, or illness severity; however, patients with language barriers, interpreter needs, and prolonged ICU/ hospital stays were less likely to attend. Language barriers and lower educational level were also associated with reduced questionnaire completion. Female sex, prolonged hospitalization, and unemployment were independently associated with worse psychological and quality of life outcomes.

### Attendance at PICS Clinic and Barriers to Follow-Up

Attendance rates in our study are consistent with previous retrospective studies that reported as few as 38% of eligible patients attended for ICU follow-up.^11^^,^^32^, ^33^^,^^32^, ^33^ In a recent systematic review, Boehm et al^8^ analyzed 15 studies assessing follow-up after ICU discharge and reported attendance rates ranging from 24% to 82%. A common reason for nonattendance is lack of awareness of the follow-up appointment. Bakhru et al^32^ showed that half of nonattenders stated they did not know about their scheduled clinic visit. Other frequently cited barriers include feeling unwell,^34^ transportation issues,^11^^,^^32^^,^^35^ financial burden, concerns about insurance,^11^ and a lack of understanding about importance of ICU follow-up and its long-term implications.^36^ Additionally, patients discharged to rehabilitation or long-term acute care facilities were less likely to return for follow-up, and demographic factors, such as being unmarried, from lower socioeconomic backgrounds, or part of underrepresented racial groups were also associated with lower attendance.^11^^,^^36^ Our study also showed some of these factors were associated with nonattendance; however, we do not know the reason patients did not attend.

Language barriers emerged as a notable challenge in our study. Although the number of affected patients was relatively small, those with LEP attended for follow-up less often than their English-speaking counterparts. Similarly, Huang et al^37^ showed that patients with language barriers are more likely to face inequities in post-COVID clinic follow-up and have worse outcomes after discharge.^37^

In contrast to retrospective studies in the literature, interventional studies that incorporated structured scheduling, patient education, and outreach to improve PICS clinics attendance found higher follow-up rates, with attendance ranging from 64% to 73% at the 3-month follow-up across nurse-led clinics, home- and phone-based recovery programs, and multidisciplinary consultations.^38^, ^39^, ^40^ These findings suggest that integrating logistical and clinical support directly into ICU follow-up interventions may significantly improve attendance.

### Patient-Reported Outcomes Among Attendees

Even after accessing follow-up clinics, patients with language barriers may face persistent inequalities. Eaton et al^16^ identified language barriers as a significant concern, noting that these patients often struggle to fully express their feelings and needs during follow-up care, and highlighted the lack of translated materials as a major limitation. In our study, we similarly observed lower completion rates for the PCL-5 and EQ-5D surveys among patients with language barriers than those among English-speaking patients, suggesting that language barriers continue to hinder effective assessment and monitoring of recovery even when patients attend follow-up appointments. However, the lack of translated resources and questionnaires in diverse languages likely explains this finding. Providing translated materials is likely to improve survey completion rates.

Furthermore, evidence suggests low SES is associated with higher rates of negative medical and socioeconomic consequences of critical illness as well as being a barrier to clinic attendance.^41^^,^^42^ A recent systematic review study showed the cumulative incidence of returning to work within 1 year of ICU discharge is lower among non-White populations, highlighting that certain racial groups may be more vulnerable. Similarly, another national US cohort study showed Hispanic and Black patients were more likely to face economic instability owing to job changes and time off work after hospitalization for COVID-19.^43^ These findings suggest that patients from racial minorities with lower SES or those with language barriers are more likely to experience hardship following critical illness. Similarly in our study, unemployed patients were more likely to be diagnosed with PTSD during follow-up clinic. However, in our study, race was not significantly associated with either quality of life or PTSD symptom scores in the analyses in our cohort.

Notably, the populations most at risk for adverse post-ICU outcomes are also the ones who face the most obstacles in accessing follow-up care, highlighting the challenges of making these clinics equally accessible and attended by those who would benefit. To address these challenges and ensure equitable ICU follow-up, it is crucial to provide dedicated, tailored follow-up care for these high-risk groups.

Previous qualitative studies have highlighted several real-world challenges in implementing ICU follow-up clinics. Haines et al^9^ conducted a study involving providers from 21 centers across 3 continents to explore the barriers and enablers of ICU follow-up care. They identified several key obstacles, including financial constraints, low patient motivation to attend, limited access to clinics, caregiver burden, out-of-pocket costs, and challenges related to an inadequately structured insurance system.^9^ “Difficulty in identifying appropriate ICU survivors who might benefit from follow-up” is another barrier to implementing ICU follow-up clinics, highlighting that even before engagement begins, it may be unclear which patients to prioritize.^9^ Although prior studies emphasize the importance of targeting patients with higher comorbidities, such as prolonged mechanical ventilation, longer ICU or hospital stays, because those are most likely to benefit, follow-up rates in this group can be as low as 20%.^44^^,^^45^ In our study, patients with longer ICU and hospital length of stay also had lower attendance rates, and although not statistically significant, patients with prolonged mechanical ventilation were also less likely to attend.

Furthermore, transportation difficulties were also identified as a barrier, and the shift to virtual ICU follow-up clinics, although potentially improving and broadening access, introduced a new challenge: digital poverty.^16^ In our virtual clinic model, approximately half of referred patients did not attend their appointment, which may be partly due to limited access to technology or internet services. However, this assumption remains speculative because we lacked specific metrics to assess the digital divide and access. We believe institutions that are facing similar language barriers should (1) engage patients with limited English proficiency earlier during hospitalization, (2) ensure interpreter needs are clearly documented and addressed before discharge, (3) provide translated educational materials about PICS and the clinic, and (4) use automated and personalized reminders in the patient’s preferred language when feasible.

### Limitations and Strengths

Our study has some limitations. The Midwest-based setting may limit generalizability to other institutional settings with diverse patient populations, referral mechanisms, and PICS infrastructure. Additionally, our 1-time follow-up model prevented long-term assessment of patient outcomes and patient trajectory and the impact of attending the PICS clinic. Furthermore, owing to the observational nature of the study and unmeasured confounding, causation cannot be established. Future studies should qualitatively evaluate the reasons behind nonattendance prospectively.

Strengths of the study include the size of our cohort. Previous ICU follow-up cohort studies included relatively small sample sizes.^34^^,^^40^ In contrast, our study included 2001 patients, substantially increasing the strength of our conclusions. In addition, this is the first study of a PICS clinic to use the novel HOUSES index as a marker of SES to understand PICS clinic attendance. Our study specifically explores the impact of language barriers on PICS clinic attendance, which has not been consistently evaluated in previous research. We hope that our findings will guide stakeholders when establishing and refining referral strategies for PICS clinics to improve attendance and accessibility for all patients.

## Conclusion

In conclusion, this retrospective study of 5 years of data from the Mayo Clinic PICS clinic shows associations between language barriers and lower attendance at the PICS clinic and, among those who did attend, lower survey completion rates. This suggests that having a language barrier may limit patients’ ability to fully engage with recovery assessments and care planning. Targeted efforts to improve outreach, provide language support, and address barriers to attendance may enhance engagement and increase the effectiveness of PICS follow-up programs for this population. Developing and providing translated surveys and educational resources is needed to improve communication, accurately assess recovery, and ensure equitable post-ICU care.

## Potential Competing Interests

The authors report no competing interests.

## Ethics Statement

The IRB reviewed and approved the study as minimal risk (IRB-24-007470).

## Declaration of AI and AI-Assisted Technologies in the Writing Process

During the preparation of this work, the authors used ChatGPT in order to refine language. After using this tool, the authors reviewed and edited the content as needed and takes full responsibility for the content of the publication.

## References

[1] Schofield-Robinson O.J., Lewis S.R., Smith A.F., McPeake J., Alderson P. (2018). Follow-up services for improving long-term outcomes in intensive care unit (ICU) survivors. Cochrane Database Syst Rev.

[2] Wiertz C.M.H., Hemmen B., Sep S.J.S. (2022). Life after COVID-19: the road from intensive care back to living—a prospective cohort study. BMJ Open.

[3] Desai S.V., Law T.J., Needham D.M. (2011). Long-term complications of critical care. Crit Care Med.

[4] Jivraj N.K., Hill A.D., Shieh M.-S. (2023). Use of mechanical ventilation across 3 countries. JAMA Intern Med.

[5] Bouzgarrou R., Farigon N., Morlat L. (2024). Incidence of post-intensive care syndrome among patients admitted to post-ICU multidisciplinary consultations: the retrospective observational PICS-MIR study. Sci Rep.

[6] Waldmann C.S. (1998). Intensive after care after intensive care. Curr Anaesth Crit Care.

[7] Brown S.M., Bose S., Banner-Goodspeed V. (2019). Approaches to addressing post-intensive care syndrome among intensive care unit survivors. A narrative review. Ann Am Thorac Soc.

[8] Boehm L.M., Potter K., McPeake J. (2024). Understanding attendance patterns and determinants in cardiac, pulmonary, and ICU Rehabilitation/Recovery programs: a systematic review and meta-analysis. Heart Lung.

[9] Haines K.J., McPeake J., Hibbert E. (2019). Enablers and barriers to implementing ICU follow-up clinics and peer support groups following critical illness: the Thrive Collaboratives. Crit Care Med.

[10] Castro-Avila A.C., Jefferson L., Dale V., Bloor K. (2021). Support and follow-up needs of patients discharged from intensive care after severe COVID-19: a mixed-methods study of the views of UK general practitioners and intensive care staff during the pandemic’s first wave. BMJ Open.

[11] Sevin C.M., Bloom S.L., Jackson J.C., Wang L., Ely E.W., Stollings J.L. (2018). Comprehensive care of ICU survivors: Development and implementation of an ICU recovery center. J Crit Care.

[12] Glimelius Petersson C., Bergbom I., Brodersen K., Ringdal M. (2011). Patients’ participation in and evaluation of a follow-up program following intensive care. Acta Anaesthesiol Scand.

[13] Ben S., Bosc R., Jiao J., Li W., Simonelli F., Zhang R. (2017).

[14] Prinjha S., Field K., Rowan K. (2009). What patients think about ICU follow-up services: a qualitative study. Crit Care.

[15] Butcher B.W., Eaton T.L., Montgomery-Yates A.A., Sevin C.M. (2022). Meeting the challenges of establishing intensive care unit follow-up clinics. Am J Crit Care.

[16] Eaton T.L., Sevin C.M., Hope A.A. (2022). Evolution in care delivery within critical illness recovery programs during the COVID-19 pandemic: a qualitative study. Ann Am Thorac Soc.

[17] Mayer K.P., Boustany H., Cassity E.P. (2020). ICU recovery clinic attendance, attrition, and patient outcomes: the impact of severity of illness, gender, and rurality. Crit Care Explor.

[18] Pandey M., Maina R.G., Amoyaw J. (2021). Impacts of English language proficiency on healthcare access, use, and outcomes among immigrants: a qualitative study. BMC Health Serv Res.

[19] Karliner L.S., Auerbach A., Nápoles A., Schillinger D., Nickleach D., Pérez-Stable E.J. (2012). Language barriers and understanding of hospital discharge instructions. Med Care.

[20] Khan A., Parente V., Baird J.D. (2022). Association of patient and family reports of hospital safety climate with language proficiency in the US. JAMA Pediatr.

[21] Bang D.W., Manemann S.M., Gerber Y. (2014). A novel socioeconomic measure using individual housing data in cardiovascular outcome research. Int J Environ Res Public Health.

[22] Cuschieri S. (2019). The STROBE guidelines. Saudi J Anaesth.

[23] Kreitner L., Crowley E., Weaver A., Wi C.I., Soma D. (2023). Evaluating the association between sociodemographic and health variables with baseline concussion testing in young athletes. Orthop J Sports Med.

[24] Kizilbash S., Wi C.I., Roy M. (2025). Socioeconomic inequities in preemptive kidney transplantation and graft survival: an innovative approach to identifying disparities in kidney transplantation. Transplant Direct.

[25] Juhn Y.J., Ryu E., Wi C.I. (2022). Assessing socioeconomic bias in machine learning algorithms in health care: a case study of the HOUSES index. J Am Med Inform Assoc.

[26] Ryu E., Olson J.E., Juhn Y.J. (2018). Association between an individual housing-based socioeconomic index and inconsistent self-reporting of health conditions: a prospective cohort study in the Mayo Clinic Biobank. BMJ Open.

[27] Stevens M.A., Beebe T.J., Wi C.I., Taler S.J., St Sauver J.L., Juhn Y.J. (2020). HOUSES index as an innovative socioeconomic measure predicts graft failure among kidney transplant recipients. Transplantation.

[28] Thacher T.D., Dudenkov D.V., Mara K.C., Maxson J.A., Wi C.I., Juhn Y.J. (2020). The relationship of 25-hydroxyvitamin D concentrations and individual-level socioeconomic status. J Steroid Biochem Mol Biol.

[29] Ryu E., Juhn Y.J., Wheeler P.H. (2017). Individual housing-based socioeconomic status predicts risk of accidental falls among adults. Ann Epidemiol.

[30] Greenwood J., Zurek K.I., Grimm J.M. (2022). Association of a housing based individual socioeconomic status measure with diabetic control in primary care practices. Prim Care Diabetes.

[31] Takahashi P.Y., Ryu E., Hathcock M.A. (2016). A novel housing-based socioeconomic measure predicts hospitalisation and multiple chronic conditions in a community population. J Epidemiol Community Health.

[32] Bakhru R., Davidson J., Bookstaver R. (2019). Implementation of an ICU recovery clinic at a tertiary care academic center. Crit Care Explor.

[33] Schwitzer E., Jensen K.S., Brinkman L. (2023). Survival ≠ recovery: a narrative review of post-intensive care syndrome. CHEST Crit Care.

[34] Stedman W., Donaldson L., Garside T. (2024). The feasibility and acceptability of a physician-led ICU follow-up service: a prospective cohort study. Aust Crit Care.

[35] Henderson P., Quasim T., Asher A. (2021). Post-intensive care syndrome following cardiothoracic critical care: feasibility of a complex intervention. J Rehabil Med.

[36] Dettling-Ihnenfeldt D.S., De Graaff A.E., Nollet F., Van Der Schaaf M. (2015). Feasibility of post-intensive care unit clinics: an observational cohort study of two different approaches. Minerva Anestesiol.

[37] Huang C.X., Okin D., Moin E.E. (2024). Post-COVID-19 clinic utilization among survivors of critical illness in two waves of SARS-CoV-2 infection. CHEST Crit Care.

[38] Cuthbertson B., Rattray J., Campbell M. (2009). The PRaCTICaL study of nurse led, intensive care follow-up programmes for improving long term outcomes from critical illness: a pragmatic randomised controlled trial. BMJ.

[39] Khan B.A., Perkins A.J., Khan S.H. (2024). Mobile critical care recovery program for survivors of acute respiratory failure: a randomized clinical trial. JAMA Netw Open.

[40] Sharshar T., Grimaldi-Bensouda L., Siami S. (2024). A randomized clinical trial to evaluate the effect of post-intensive care multidisciplinary consultations on mortality and the quality of life at 1 year. Intensive Care Med.

[41] Rodriguez-Gutierrez R., Herrin J., Lipska K.J. (2019). Racial and ethnic differences in 30-day hospital readmissions among US adults with diabetes. JAMA Netw Open.

[42] Chu J.N., Wong J., Bardach N.S. (2024). Association between language discordance and unplanned hospital readmissions or emergency department revisits: a systematic review and meta-analysis. BMJ Qual Saf.

[43] Admon A.J., Iwashyna T.J., Kamphuis L.A. (2023). Assessment of symptom, disability, and financial trajectories in patients hospitalized for COVID-19 at 6 months. JAMA Netw Open.

[44] Nakanishi N., Liu K., Hatakeyama J. (2024). Post-intensive care syndrome follow-up system after hospital discharge: a narrative review. J Intensive Care.

[45] Teixeira C., Rosa R.G. (2018). Post-intensive care outpatient clinic: is it feasible and effective?. A literature review.

